# Stroke-heart syndrome and early mortality in patients with acute ischaemic stroke using hierarchical cluster analysis: An individual patient data pooled analysis from the VISTA database

**DOI:** 10.1177/23969873241290440

**Published:** 2024-10-13

**Authors:** Hironori Ishiguchi, Bi Huang, Wahbi K. El-Bouri, Gregory Y. H. Lip, Azmil H. Abdul-Rahim

**Affiliations:** 1 Liverpool Centre for Cardiovascular Science at University of Liverpool, Liverpool John Moores University and Liverpool Heart & Chest Hospital, Liverpool, UK; 2 Division of Cardiology, Department of Medicine and Clinical Science, Yamaguchi University Graduate School of Medicine, Ube, Japan; 3 Department of Cardiovascular and Metabolic Medicine, Institute of Life Course and Medical Sciences, University of Liverpool, Liverpool, UK; 4 Danish Centre for Health Services Research, Department of Clinical Medicine, Aalborg University, Aalborg, Denmark; 5 Stroke Division, Department Medicine for Older People, Mersey and West Lancashire Teaching Hospitals NHS Trust, Prescot, UK

**Keywords:** Hierarchical cluster analysis, stroke-heart syndrome, ischaemic stroke

## Abstract

**Background:**

The patient clinical phenotypes at particularly high risk for early cardiac complications after a recent acute ischaemic stroke (AIS), that is, stroke-heart syndrome (SHS), remain poorly defined. We utilised hierarchical cluster analysis to identify specific phenotypic profiles associated with this risk.

**Methods:**

We gathered data on patients with AIS from the Virtual International Stroke Trials Archive, a global repository of clinical trial data. We examined cardiac complications within 30 days post-stroke, including acute coronary syndrome, heart failure, arrhythmias and cardiorespiratory arrest. We employed hierarchical cluster analysis to define distinct phenotypic risk profiles. The incidence/risk of SHS and 90-day mortality were compared across these profiles.

**Results:**

We included 12,482 patients (mean age 69 ± 12 years; 55% male), yielding five phenotypes: Profile 1 (‘*elderly and AF*’), Profile 2 (‘*young and smoker*’), Profile 3 (‘*young*’), Profile 4 (‘*cardiac comorbidities*’) and Profile 5 (‘*hypertension with atherosclerotic comorbidities*’). Profiles 4 and 1 exhibited the highest risk for SHS (adjusted HR (95% CI): 2.01 (1.70–2.38) and 1.26 (1.05–1.51), respectively, compared to Profile 3), while Profiles 5 and 2 showed moderate risk and Profile 3 had the lowest risk. Although Profiles 1 and 4 were at the highest risk for most SHS presentations, Profile 5 had the highest risk for cardiorespiratory arrest (adjusted HR (95% CI): 2.99 (1.22–7.34)). The 90-day mortality risk was stratified by phenotype, with the highest risk observed in Profiles 5, and 4.

**Conclusions:**

Hierarchical cluster analysis effectively identified phenotypes with the highest risk of SHS and early mortality in patients with AIS.

## Graphical abstract

**Graphical abstract:**
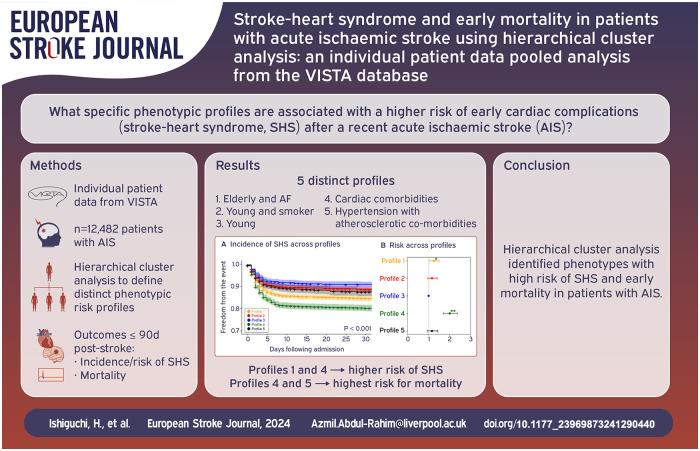

## Introduction

Cardiac complications are a common cause of morbidity and mortality following acute ischaemic stroke (AIS).^1^ The cardiac complications also adversely affect long-term prognosis.^2,3^

Stroke-heart syndrome (SHS) comprehensively refers to cardiac complications, either new or worsening of pre-existing heart diseases, occurring within 30 days following the onset of AIS.^4,5^ The presentations of SHS include acute myocardial infarction (MI)/acute myocardial injury, heart failure (HF) or left ventricular (LV) dysfunction (including Takotsubo syndrome), arrhythmias (including atrial fibrillation (AF) and electrocardiogram (ECG) abnormalities) and sudden cardiac death. The pathophysiology of SHS is thought to result from brain-heart interactions, specifically stroke-induced cerebral damage, through autonomic dysregulation, neurohormonal disturbances and abnormal neuroinflammatory responses.^6,7^

Regardless of ischaemic stroke aetiology, there appear to be distinct phenotypes of patients with AIS.^8–10^ Hierarchical cluster analysis allows categorisation of heterogeneous populations into distinct clusters based on shared phenotypes.^11^ Using this approach, we aimed to identify specific phenotypic profiles associated with the risk of SHS and mortality in patients with AIS.

## Method

### Data resource

We conducted a retrospective analysis of individual patient data pooled from randomised clinical trials within the Virtual International Stroke Trials Archive (VISTA), URL: https://www.virtualtrialsarchives.org/vista/.^12^ VISTA serves as a collaborative platform, aggregating anonymised patient data from completed acute stroke trials (spanning from 1998 to 2010) for novel exploratory analyses. We selected patients with ischaemic stroke who were randomised to receive either a placebo or a drug now known to have no confirmed effect on stroke outcomes. We included patients for whom baseline demographic and outcome information was available. All stroke patients received treatment according to institutional practices and stroke guidelines that were acceptable at the time of the trial. The VISTA data used in this analysis did not include trials specifically testing thrombolysis therapy. However, thrombolysis was administered as standard therapy, where appropriate, within the included trial. Our analysis was conducted and reported in accordance with the Strengthening the Reporting of Observational Studies in Epidemiology (STROBE) guidelines for cohort studies.^13^

### Study design

We included patients who presented with AIS within 24 h of stroke onset. The primary outcome was the development of SHS and each of its individual presentations. The secondary outcomes were mortality and poor functional outcome using the modified Rankin Scale at 90 days.

### Stroke-heart syndrome

SHS was characterised as patients who exhibited any of the following cardiac presentations within 30 days post-onset of AIS: acute coronary syndrome (ACS, encompassing acute MI and unstable angina)/myocardial injury (typically coded as ‘elevated cardiac enzymes’ or ‘troponin increase’), HF/LV dysfunction, AF/atrial flutter (AFL), other arrhythmia/ECG abnormalities (typically coded as ‘QT prolongation’) and cardiorespiratory arrest.

The identification of these cardiac events was based on a thorough review of adverse events listed in the study database. To ensure the reliability of data collection, two researchers (HI and BH) independently gathered data and subsequently cross-checked their results to confirm accuracy and consistency.

### Hierarchical cluster analysis

We utilised Ward’s minimum variance method to perform a hierarchical cluster analysis, focusing on a demographic dataset comprising 12 key variables. We selected key variables that are clinically important risk factors for stroke and the development of cardiac complications, such as age and stroke severity. Additionally, we prioritised variables with low missing values, less than 20% within the VISTA database. The variables included age, sex, initial score on the National Institutes of Health Stroke Scale (NIHSS), the use of intravenous thrombolysis, systolic and diastolic blood pressure, smoking status, and comorbidities. The comorbidities included hypertension, diabetes, history of stroke, AF and MI. Missing values were imputed by Multiple Imputation by Chained Equations method.

### Statistical analysis

Variables with normal distributions were represented as mean ± standard deviation, whereas those with non-normal distributions were presented as medians with their respective first and third quartiles. Categorical variables were expressed as counts with proportions. Analysis of variance was utilised to compare these variables. Event-free survival rates for each assessed outcome were expressed using Kaplan-Meier curve. The log-rank test was employed to compare of differences across clusters. To intuitively visualise the difference across clusters, we translated the cumulative incidence into hazard ratios (HR) for each outcome, along with their 95% confidence intervals (CI) using a univariate Cox proportional hazards model. The hazard ratios were adjusted for age and sex throughout the analysis. The cluster with the minimal event incidence was used as the reference point. The mRS distribution at 90 days across the profiles was compared using analysis of variance. A *p*-value below 0.05 was set as the threshold for statistical significance. All statistical evaluations were performed using R software, version 4.3.0, on a trusted research network provided by VISTA.

## Results

### Patient demographics of each profile

**Figure 1.**
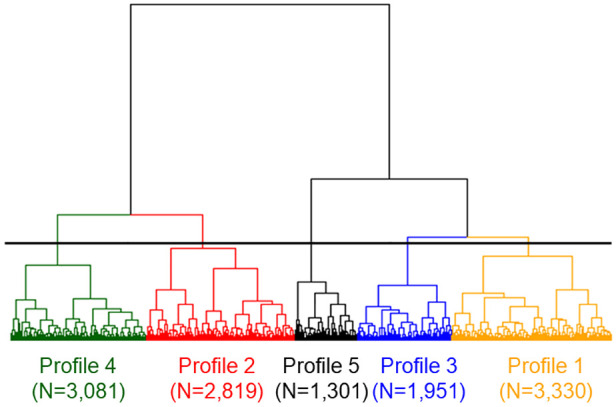
Dendrogram obtained from hierarchical cluster analysis. The horizonal line indicates the cut-off line the whole cohort dividing five profiles.

From an initial cohort of 16,095 patients with AIS, 12,482 were deemed eligible for inclusion in this study (Supplemental Figure 1). Based on dendrogram analysis, the cohort was divided into five distinct profiles, as depicted in Figure 1. The demographic characteristics of each profile are summarised in Table 1.

**Table 1.** Patient demographics.

Variables	Profile 1 (*N* = 3330)	Profile 2 (*N* = 2819)	Profile 3 (*N* = 1951)	Profile 4 (*N* = 3081)	Profile 5 (*N* = 1301)	*p*-Value
Age (years), mean ± SD [0]	76 ± 9	66 ± 13	64 ± 11	68 ± 14	70 ± 11	<0.001
Male, *n* (%) [0]	1599 (48)	1779 (63)	1233 (63)	1602 (52)	708 (54)	0.054
History of stroke, *n* (%) [0.3]	877 (26)	620 (22)	393 (20)	674 (22)	360 (28)	0.160
Baseline NIHSS, mean ± SD [14]	13 ± 6	12 ± 6	12 ± 5	14 ± 6	12 ± 5	0.082
Use of IV thrombolysis, *n* (%) [5.4]	909 (27)	714 (25)	407 (21)	948 (31)	229 (18)	0.023
Systolic BP (mmHg), mean ± SD [0.7]	172 ± 12	143 ± 8	164 ± 10	129 ± 14	198 ± 16	<0.001
Diastolic BP (mmHg), mean ± SD [0.7]	82 ± 11	85 ± 8	97 ± 8	68 ± 11	106 ± 12	<0.001
Hypertension, *n* (%) [6]	2529 (76)	1755 (62)	1364 (70)	1767 (57)	1048 (81)	<0.001
Diabetes, *n* (%) [0.04]	813 (24)	561 (20)	396 (20)	596 (19)	315 (24)	0.020
History of AF, *n* (%) [11]	977 (29)	639 (23)	411 (21)	804 (26)	301 (23)	<0.001
History of MI, *n* (%) [18]	413 (12)	408 (14)	213 (11)	544 (18)	139 (11)	0.036
Smoking status, *n* (%) [3]						<0.001
Never smoked	1552 (47)	1101 (39)	775 (40)	1233 (40)	541 (42)	
Current smoker	1282 (38)	880 (31)	569 (29)	1084 (35)	473 (36)	
Ex-smoker	496 (15)	837 (30)	607 (31)	763 (25)	286 (22)	

AF: atrial fibrillation; BP: blood pressure; IV: intravenous; MI: myocardial infarction; NIHSS: National Institutes of Health Stroke Scale; SD: standard deviation.

Numerical data are expressed as mean ± SD or median (interquartile range; first quartile, third quartile). Categorical data are expressed as percentages and numbers. [] indicates the missing rate (%).

Profile 1, distinguished by its association with ‘elderly and AF’, exhibited the highest mean age at 76 ± 9 years, along with a significant prevalence of AF at 29%. This profile also showed the second highest systolic blood pressure at 172 ± 12 mmHg and a history of stroke in 26%, both second only to Profile 5. Additionally, Profile 1 was characterised by a majority female population (52%).

Profile 2, labelled as ‘young and smoker’ features a relatively young mean age of 66 ± 13 years, the second youngest following Profile 3. This profile had the highest combined rate of current/ex-smokers, totalling 61%. Additionally, it shared the highest proportion of males (63%) with Profile 3. The baseline NIHSS score was the lowest in this group, at 12 ± 6, equal to that of Profile 3. Moreover, the incidence of previous history of MI was the second highest at 14%, compared to Profile 4.

Profile 3, labelled as the ‘young’ group, comprised the youngest cohort with a mean age of 64 ± 11 years and was predominantly male (63%). This group was characterised by the lowest baseline NIHSS score (12 ± 5) and the smallest proportion of individuals with a history of stroke (20%). Additionally, it displayed the lowest prevalence of cardiac comorbidities, including AF at 21% and MI at 11%.

Profile 4, identified as the ‘cardiac comorbidities’ group, was distinguished by the highest prevalence of MI history at 18%, and the second highest prevalence of AF at 26%, following Profile 1. The profile also had the highest NIHSS, followed by Profile 1 (‘elderly and AF’). Additionally, it had the second highest female representation at 48%, after Profile 1. The group showed the lowest systolic and diastolic blood pressure among all profiles, at 129 ± 14 mmHg and 68 ± 11 mmHg, respectively.

Profile 5, designated as ‘hypertension with atherosclerotic comorbidities’ was characterised by the highest prevalence of hypertension at 81% and the most elevated mean systolic and diastolic blood pressure, at 198 ± 16 mmHg and 106 ± 12 mmHg, respectively. Additionally, this group displayed the highest percentages of those with a history of stroke (28%) and diabetes (24%).

### Incidence of stroke-heart syndrome

In the entire dataset, 248 out of 6548 adverse event terms were identified as cardiac events. A total of 1774 patients (1997 events; 14.2% overall) experienced SHS. The distribution of these presentations was as follows: other arrhythmia/ECG abnormalities were the most common, accounting for 53% of cases, followed by AF/AFL at 34%, HF/LV dysfunction at 18%, ACS/myocardial injury at 8% and cardiorespiratory arrest at 4.5%.

Among patients who developed ACS/myocardial injury, 91% were classified as ACS and 9% as myocardial injury. Similarly, among patients who developed the HF/LV dysfunction, 92% of patients were identified as having HF, while 8% were classified as having LV dysfunction. Additionally, 94% of AF/AFL cases were classified as AF, and 95% of other arrhythmia/ECG abnormalities cases were categorised as other arrhythmia.

Among patients who developed AF/AFL within 30 days of stroke, 75% had no prior history of AF (i.e. de novo AF). Similarly, 67% of those who developed heart HF/LV dysfunction were de novo HF cases.

Among patients with ‘other arrhythmias/ECG abnormalities’, the largest arrhythmia subcategory was bradyarrhythmia (37%), followed by ventricular arrhythmias (17%). While most arrhythmias subcategories were comparable across profiles, the proportion of supraventricular arrhythmias, excluding AF/AFL, was significantly higher in Profile 5 (Supplemental Table 1). In patients with ECG abnormalities, the most common findings were ST-T changes (39%) and QT prolongation (25%).

### Incidence of stroke-heart syndrome across the profiles

**Figure 2.**
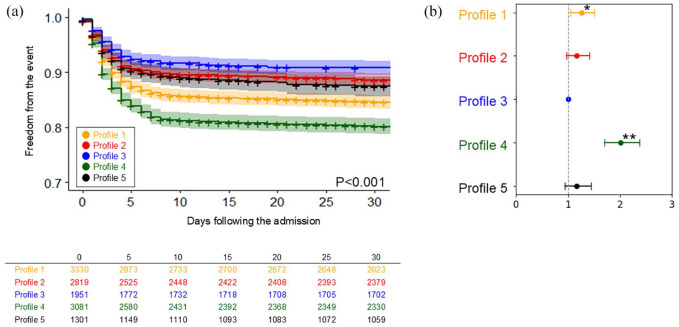
The comparison of the incidence of stroke-heart syndrome across five profiles: (a) The Kaplan-Meier curve and (b) Plot of hazard ratios adjusted for age and sex, with 95% confidence intervals. SHS: stroke-heart syndrome. Asterisk indicates statistical significance (*p < 0.05, **p < 0.001).

Figure 2 and Supplemental Table 2 compare the cumulative incidence of SHS development across the five different profiles. Profiles 1 (‘elderly and AF’) and 4 (‘cardiac comorbidities’) demonstrated the highest incidence. Profiles 2 (‘young and smoker’) and 5 (‘hypertension with atherosclerotic comorbidities’) showed moderate incidence, while Profile 3 (‘young’) exhibited with the lowest incidence.

The adjusted HRs across the profiles are detailed in Table 2. Compared with Profile 3 (‘young’), Profile 1 and 4 exhibited a significantly higher risk for SHS.

**Table 2.** Comparison of risks of stroke-heart syndrome, each presentation, and mortality across the profiles.

Profiles	HR (95% CI)	*p*-Value
SHS		
Profile 1*	1.26 (1.05−1.51)	0.009
Profile 2	1.17 (0.97−1.41)	0.084
Profile 3	1 (Reference)	
Profile 4**	2.01 (1.70−2.38)	<0.001
Profile 5	1.17 (0.94−1.45)	0.145
ACS/myocardial injury		
Profile 1	1.06 (0.57−1.96)	0.834
Profile 2	1.30 (0.71−2.38)	0.382
Profile 3	1 (Reference)	
Profile 4	1.71 (0.96−3.03)	0.067
Profile 5	1.02 (0.47−2.16)	0.960
HF/LV dysfunction		
Profile 1	0.72 (0.48−1.10)	0.133
Profile 2	0.98 (0.64−1.50)	0.933
Profile 3	1 (Reference)	
Profile 4*	1.52 (1.03−2.25)	0.034
Profile 5	1.01 (0.60−1.61)	0.974
AF/AFL		
Profile 1	1.29 (0.94−1.77)	0.105
Profile 2	1.16 (0.83−1.62)	0.359
Profile 3	1 (Reference)	
Profile 4**	2.05 (1.51−2.78)	<0.001
Profile 5	1.15 (0.78−1.69)	0.462
Other arrhythmia/ECG abnormalities		
Profile 1*	1.43 (1.12−1.82)	0.004
Profile 2	1.13 (0.87−1.46)	0.346
Profile 3	1 (Reference)	
Profile 4**	2.29 (1.82−2.89)	<0.001
Profile 5	1.18 (0.87−1.60)	0.274
Cardiorespiratory arrest		
Profile 1	1.11 (0.45−2.75)	0.805
Profile 2	1.20 (0.48−3.03)	0.689
Profile 3	1 (Reference)	
Profile 4	2.11 (0.91−4.91)	0.080
Profile 5*	2.99 (1.22−7.34)	0.016
90-day mortality		
Profile 1	1.18 (0.97−1.45)	0.092
Profile 2	1.17 (0.95−1.44)	0.134
Profile 3	1 (Reference)	
Profile 4**	1.40 (1.14−1.71)	<0.001
Profile 5*	1.44 (1.14−1.82)	0.001

ACS: acute coronary syndrome; AF: atrial fibrillation; AFL: atrial flutter; CI: confidence interval; ECG: electrocardiogram; HF: heart failure; HR: hazard ratio; LV: left ventricular; SHS: stroke-heart syndrome.

Asterisks indicates statistical significance (*p < 0.05, **p < 0.001).

### Incidence of each SHS presentations across the profiles

**Figure 3.**
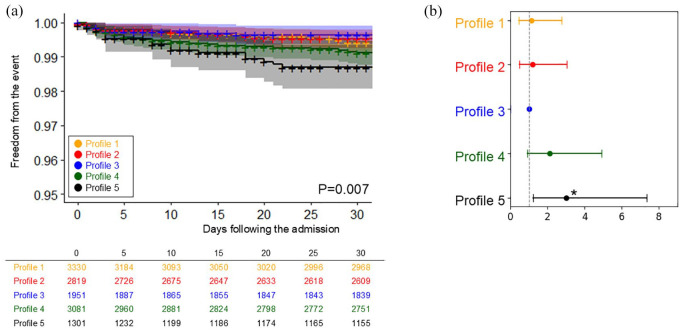
The comparison of the incidence of cardiorespiratory arrest across five profiles: (a) The Kaplan-Meier curve and (b) plot of hazard ratios adjusted for age and sex, with 95% confidence intervals. Asterisk indicates statistical significance (*p < 0.05).

**Figure 4.**
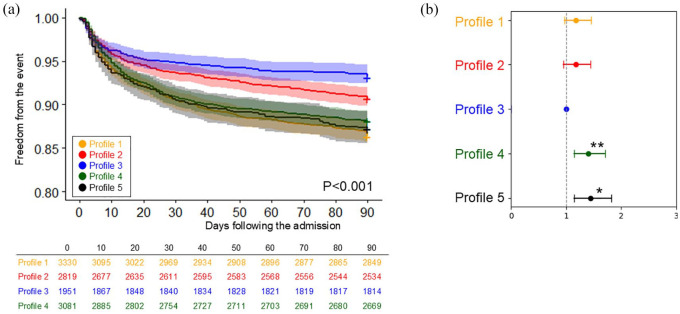
The comparison of the incidence of mortality across five profiles: (a) The Kaplan-Meier curve and (b) Plot of hazard ratios adjusted for age and sex, with 95% confidence intervals. Asterisk indicates statistical significance (*p < 0.05, **p < 0.001).

Figures 3 and 4 and Supplemental Figures 2 to 5 compare the cumulative incidence of each of the SHS presentations across the five different profiles.

For ACS/myocardial injury, Profile 4 (‘cardiac comorbidities’) demonstrated the highest incidence (Supplemental Figure 2), compared to Profile 3 (‘young’) which was the lowest (HR (95% CI): 1.71 (0.96–3.03), *p* = 0.067), although this did not reach statistical significance. The incidences among the other profiles were relatively comparable.

For patients who developed HF/LV dysfunction, Profiles 4 (‘cardiac comorbidities’) maintained the highest incidence (cumulative incidence freedom from the event (95% CI): 96.3% (95.6–96.9), *p* < 0.001), a similar trend to that observed in ACS/myocardial injury (Supplemental Figure 3). Profiles 1 (‘elderly and AF’) and 5 (‘hypertension with atherosclerotic comorbidities’) presented the second highest incidences.

In cases with AF/AFL and other arrhythmia/ECG abnormalities, both presentations exhibited a similar pattern (Supplemental Figures 4 and 5). Profiles 1 (‘elderly and AF’) and 4 (‘cardiac comorbidities’) recorded the highest incidences among all profiles. This was followed by Profile 5 (‘hypertension with atherosclerotic comorbidities’), Profile 2 (‘young and smoker’) and Profile 3 (‘young’).

For cardiorespiratory arrest, the pattern was markedly different from other SHS presentations. As detailed in Figure 3, Profile 5 (‘hypertension with atherosclerotic comorbidities’) exhibited the highest incidence of cardiorespiratory arrest across all profiles (cumulative incidence freedom from the event (95% CI): 87.2% (85.4–89.1), *p* < 0.001). This was sequentially followed by Profile 4 (‘cardiac comorbidities’), and Profile 1 (‘elderly and AF’).

### Ninety-day mortality and mRS at 90 days

The cumulative incidence of mortality within 90 days is presented in Figure 4. Profiles 1 (‘elderly and AF’), 4 (‘cardiac comorbidities’) and 5 (‘hypertension with atherosclerotic comorbidities’) exhibited the highest mortality rates. These were followed by Profile 2 (‘young and smoker’) and Profile 3 (‘young’). When compared to Profile 3, all other profiles tended to exhibit higher risks. The mRS at 90 days also followed similar trend, with Profiles 1 (mRS 3.2 ± 1.9) and 5 (mRS 3.0 ± 1.9) exhibiting the highest scores, and Profile 3 had the lowest (mRS 2.5 ± 1.8), as shown in Supplemental Table 3.

## Discussion

The key findings of our study can be summarised as follows (Visual Abstract). First, using hierarchical cluster analysis, we effectively stratified patients with AIS into distinct phenotypes based on their risk of developing SHS. Profiles 4 (‘cardiac comorbidities’) and 1 (‘elderly and AF’) showed the highest risk, Profiles 5 (‘hypertension with atherosclerotic comorbidities’) and 2 (‘young and smoker’) demonstrated moderate risk and Profile 3 (‘young’) had the lowest risk. Second, while Profiles 1 (‘elderly and AF’) and 4 (‘cardiac comorbidities’) showed the highest risk for most SHS presentations, Profile 5 (‘hypertension with atherosclerotic comorbidities’) exhibited the highest risk for cardiorespiratory arrest. Third, the mortality risk within 90 days was also stratified by the phenotypes, with the highest risk observed in Profiles 1 (‘elderly and AF’), 5 (‘hypertension with atherosclerotic comorbidities’) and 4 (‘cardiac comorbidities’), a moderate risk in Profile 2 (‘young and smoker’), and the lowest risk in Profile 3 (‘young’).

### Phenotypic features in ischaemic stroke

Multimorbidity is highly prevalent among patients with ischaemic stroke and has been reported to adversely affect outcomes in terms of both mortality and functional prognosis.^9,14^ It also contributes to the aetiological heterogeneity of the ischaemic stroke.^9^

To address this complexity and stratify risk effectively, previous studies have employed cluster analysis to delineate distinct risk profiles.^8,9,15^ Sennfält et al.^9^ identified risk profiles predominantly characterised by (i) ‘non-cardiovascular comorbidities’, including dementia and malignancy, (ii) ‘AF’, (iii) ‘atherosclerotic risk factors’ and (iv) ‘cardiovascular comorbidities’, excluding AF. Akyea et al. revealed that risk profiles encompassing elderly individuals with a concomitant presence of ‘arrhythmic comorbidities’ and ‘dementia’ were linked to significantly worse outcomes. In contrast, profiles characterised by ‘younger individuals’, who tended to be current smokers with fewer comorbidities, displayed a relatively better prognosis.^15^

While our study did not evaluate non-cardiovascular comorbidities, our findings align with these studies, identifying similar risk profiles such as ‘elderly and AF’, ‘young’ and ‘cardiac comorbidities’. In particular, our data indicate that the profile consisting of elderly individuals with AF (Profile 1, ‘elderly and AF’) exhibited the highest mortality risk. Conversely, profiles of younger individuals with fewer comorbidities demonstrated the lowest mortality risk, consistent with previous literature. Our research contributes new insights by revealing that the Profile 1 (‘elderly and AF’) not only faces increased mortality risk but also a heightened risk of cardiac complications post-stroke.

### Risk profiles for stroke-heart syndrome

Prior research has demonstrated risk factors and typical profiles (e.g. elderly females for Takotsubo syndrome^5^) associated with the development of cardiac complications in patients with ischaemic stroke.

For severe cardiac complications post-stroke such as acute MI and HF, cardiovascular comorbidities (e.g. chronic HF and history of coronary heart disease), high burden of atherosclerotic risk factors and high stroke severity have been considered the primary risk factors.^16–18^ Additionally, a stroke anatomical lesion that involving the central autonomic network, particularly the insular cortex, may influence the development of arrhythmia and ECG abnormalities.^19^

Our findings are consistent with previous research, as the profiles identified with a high risk of SHS (‘elderly and AF’ and ‘cardiac comorbidities’) were characterised by a high prevalence of cardiovascular comorbidities, such as MI and AF, as well as elevated stroke severity, as indicated by higher scores on the NIHSS.

Meanwhile, the risk profiles associated with cardiorespiratory arrest remain underexplored due to their rare occurrence. The potential link between insular involvement and cardiorespiratory arrest continues to be controversial.^20,21^ Our research introduces several novel findings, indicating that Profile 5 (‘hypertension with atherosclerotic comorbidities’) particularly suffered an increased risk of cardiorespiratory arrest, setting it apart from other major presentations of SHS. This suggests a possible unique relationship between hypertension at the onset and the development of cardiorespiratory arrest during the acute period. Furthermore, it suggests that the mechanisms leading to cardiorespiratory arrest may be distinct from those causing other SHS presentations. However, due to the limited available data, further research is warranted to validate these findings and elucidate the underlying mechanisms.

### Clinical implications

Our study highlights the utility of hierarchical cluster analysis in profiling to identify risks associated with the development of SHS and mortality. Specifically, profiles with a high incidence of SHS, such as Profiles 1 (‘elderly and AF’) and 4 (‘cardiac comorbidities’) also exhibited a heightened risk of mortality. Recognising such profiles could be invaluable in clinical settings for effective risk management.

In contrast, Profile 5 (‘hypertension with atherosclerotic comorbidities’) demonstrated a significant mortality risk, despite a moderate incidence of SHS. This increased risk may be attributed to the profile’s distinct susceptibility to cardiorespiratory arrest. The heightened risk of cardiorespiratory arrest in this profile underscores the need for clinical vigilance.

### Limitations

Our study is subject to several limitations. First, the retrospective nature of our data analysis resulted in the absence of certain crucial variables, limiting our hierarchical cluster analysis primarily to basic demographic data. This approach excluded detailed factors such as anatomical lesion involvement or specific ischaemic stroke mechanisms, which could potentially enhance the identification of high-risk profiles, if included. Second, the detection of cardiac events was dependent on the documentation of adverse events across various studies compiled within the VISTA database, rather than through direct review of medical records. This may have led to an underestimation of the actual incidence of SHS and its individual presentations. Third, our study population consists of cohorts from randomised controlled trials, which may differ from the general acute stroke population, potentially limiting the generalisability of our findings. As such, validation in real-world cohorts is necessary to confirm the applicability of our results. Forth, we do not have data on cardiovascular mortality at 90 days. Although profiles with higher cardiac events rates also demonstrated higher all-cause mortality, we are unable to establish a causal relationship between cardiac events and mortality.

## Conclusions

Certain phenotypic profiles identified through hierarchical cluster analysis are associated with the highest risk of SHS and early mortality in patients with recent ischaemic stroke. These profiles may inform clinical management and risk stratification for patients.*VISTA-Acute Steering Committee members: K.R. Lees (Chair), A. Alexandrov, P.M. Bath, E. Bluhmki, N. Bornstein, C. Chen, L. Claesson, J. Curram, S.M. Davis, H-C. Diener, G. Donnan, M. Fisher, M. Ginsberg, B. Gregson, J. Grotta, W. Hacke, M.G. Hennerici, M. Hommel, M. Kaste, P. Lyden, J. Marler, K. Muir, C. Roffe, R. Sacco, A .Shuaib, P. Teal, N. Venketasubramanian, N.G. Wahlgren, and S. Warach.

## Supplemental Material

sj-docx-1-eso-10.1177_23969873241290440 – Supplemental material for Stroke-heart syndrome and early mortality in patients with acute ischaemic stroke using hierarchical cluster analysis: An individual patient data pooled analysis from the VISTA database Supplemental material, sj-docx-1-eso-10.1177_23969873241290440 for Stroke-heart syndrome and early mortality in patients with acute ischaemic stroke using hierarchical cluster analysis: An individual patient data pooled analysis from the VISTA database by Hironori Ishiguchi, Bi Huang, Wahbi K. El-Bouri, Gregory Y. H. Lip and Azmil H. Abdul-Rahim in European Stroke Journal
