# Supplementary material for: Stroke-heart syndrome and early mortality in patients with acute ischaemic stroke using hierarchical cluster analysis: An individual patient data pooled analysis from the VISTA database
Source: Eur Stroke J. 2024 Oct 13;10(2):478–86. doi: 10.1177/23969873241290440 (PMC11556556; doi:10.1177/23969873241290440)
Supplement: sj-docx-1-eso-10.1177_23969873241290440 – Supplemental material for Stroke-heart syndrome and early mortality in patients with acute ischaemic stroke using hierarchical cluster analysis: An individual patient data pooled analysis from the VISTA database [file sj-docx-1-eso-10.1177_23969873241290440.docx]

**Supplementary Materials**

**Supplementary Figure 1.** Study Diagram

**Supplementary Figure 2.** The Comparison of the Incidence of Acute Coronary Syndrome/ Myocardial Injury Across Five Profiles

**Supplementary Figure 3.** The Comparison of the Incidence of Heart Failure/ Left Ventricular Dysfunction Across Five Profiles

**Supplementary Figure 4.** The Comparison of the Incidence of Atrial Fibrillation/ Atrial Flutter Across Five Profiles

**Supplementary Figure 5.** The Comparison of the Incidence of Other Arrhythmia/ Electrocardiogram Abnormalities Across Five Profiles

**Supplementary Table 1.** The Comparison of Arrhythmia Subcategories across Profiles

**Supplementary Table 2.** Cumulative Incidence Freedom from the Each Clinical Event across Profiles

**Supplementary Table 3.** Modified Rankin Scale at Ninety-Day across Profiles

**Supplementary Figure 1. Study Diagram**


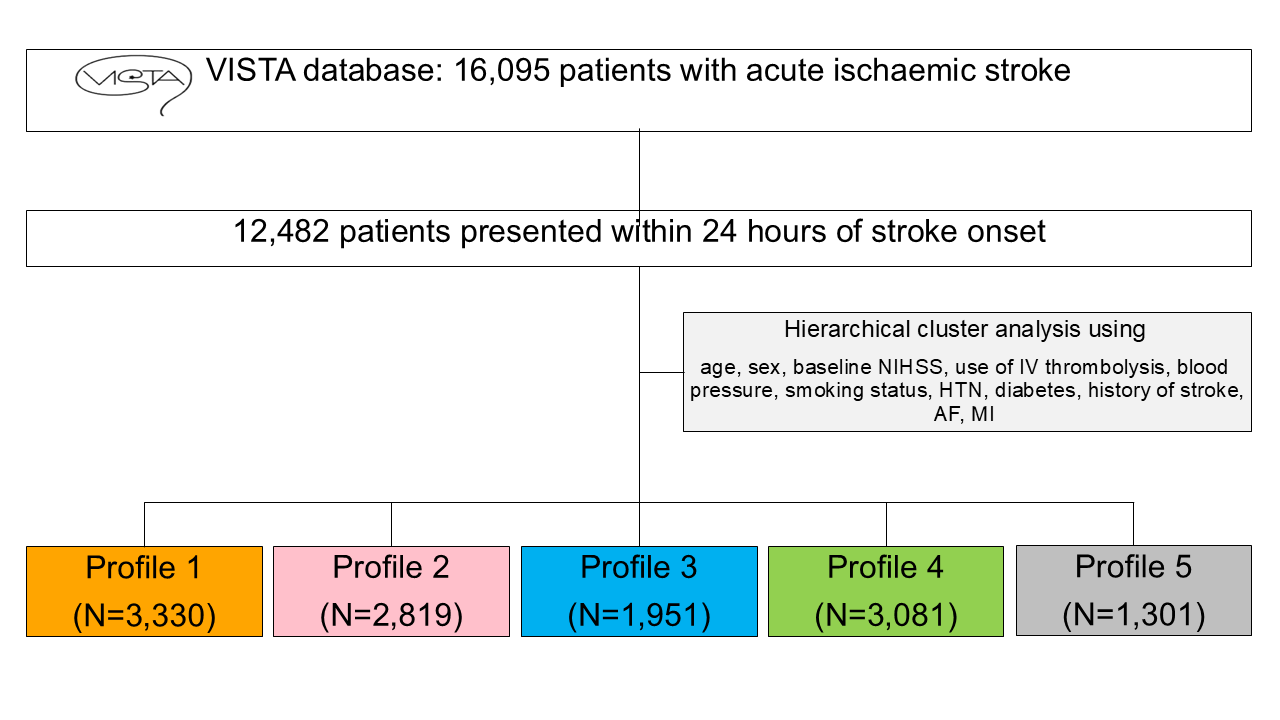


AF, atrial fibrillation; HTN, hypertension; IV, intravenous; MI, myocardial infarction; NIHSS, National Institutes of Health Stroke Scale

**Supplementary Figure 2. The Comparison of the Incidence of Acute Coronary Syndrome/ Myocardial Injury Across Five Profiles**


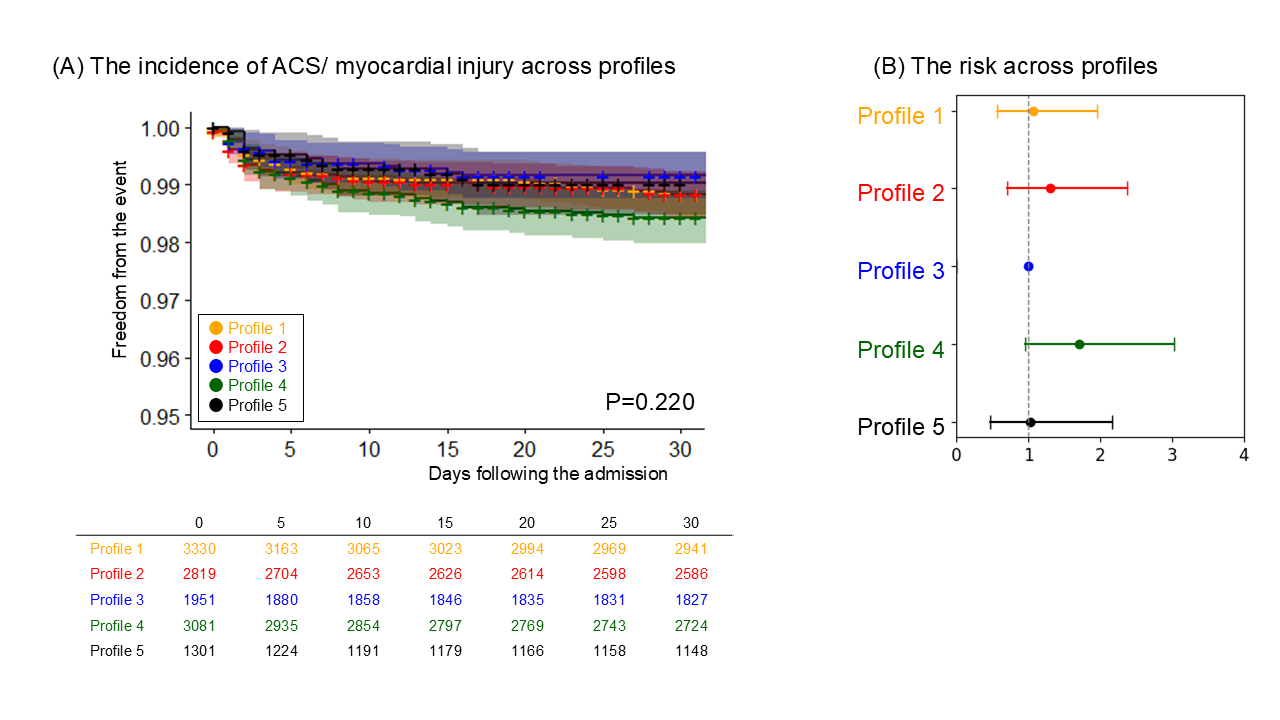


(A) The Kaplan-Meier curve. (B) Plot of hazard ratios adjusted for age and sex, with 95% confidence intervals.

ACS, acute coronary syndrome

**Supplementary Figure 3. The Comparison of the Incidence of Heart Failure/ Left Ventricular Dysfunction Across Five Profiles**


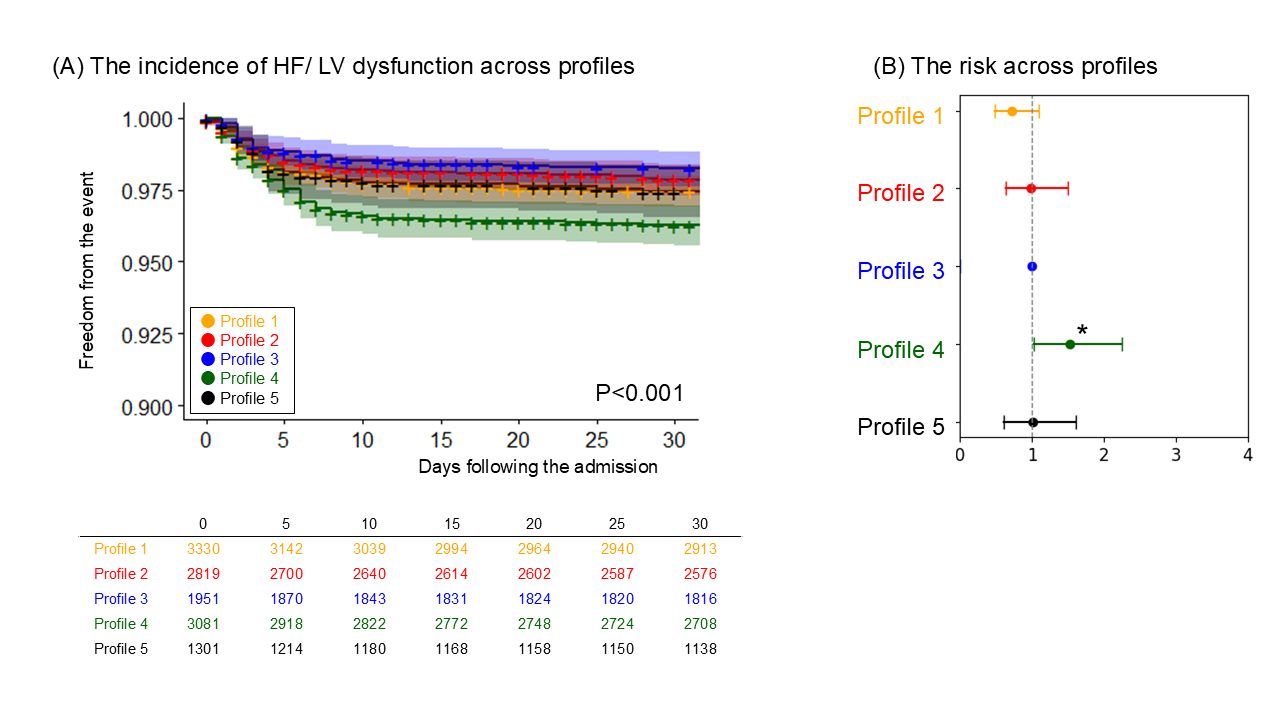


(A) The Kaplan-Meier curve. (B) Plot of hazard ratios adjusted for age and sex, with 95% confidence intervals.

HF, heart failure; LV, left ventricular. Asterisk indicates statistical significance (*P<0.05).

**Supplementary Figure 4. The Comparison of the Incidence of Atrial Fibrillation/ Atrial Flutter Across Five Profiles**


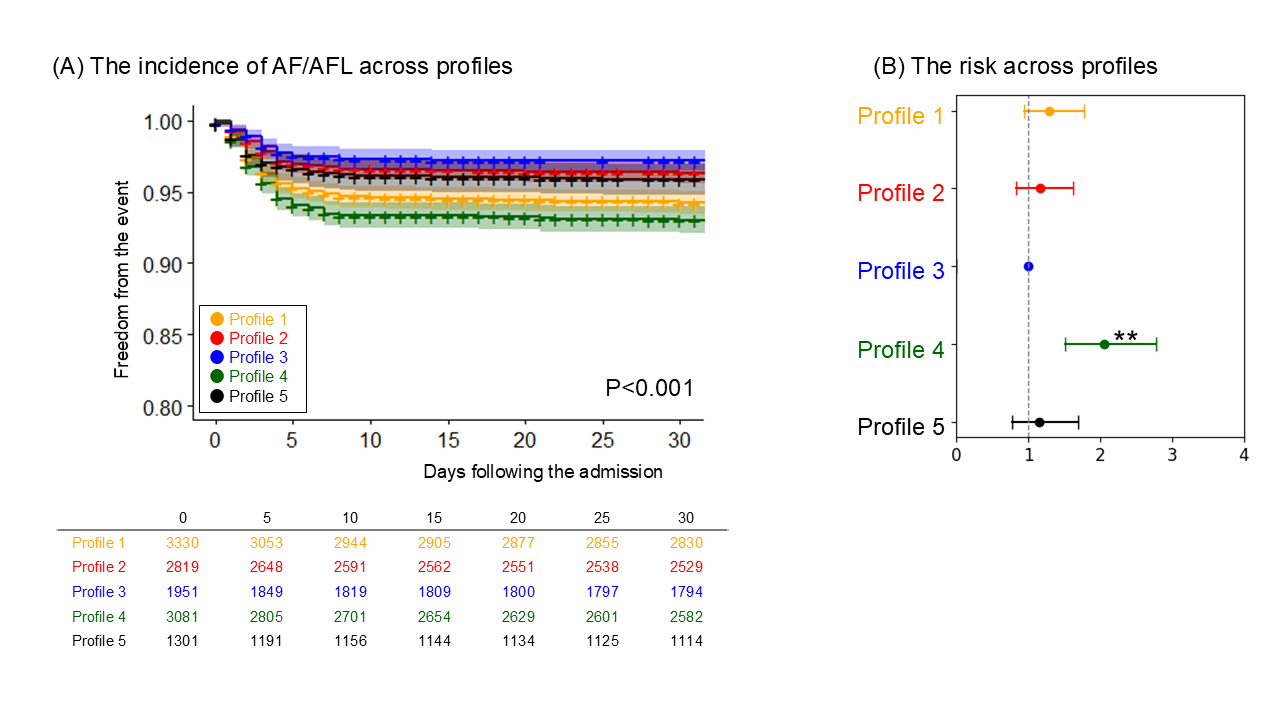


(A) The Kaplan-Meier curve. (B) Plot of hazard ratios adjusted for age and sex, with 95% confidence intervals.

AF, atrial fibrillation; AFL, atrial flutter. Asterisk indicates statistical significance (**P<0.001).

**Supplementary Figure 5. The Comparison of the Incidence of Other Arrhythmia/ Electrocardiogram Abnormalities Across Five Profiles**


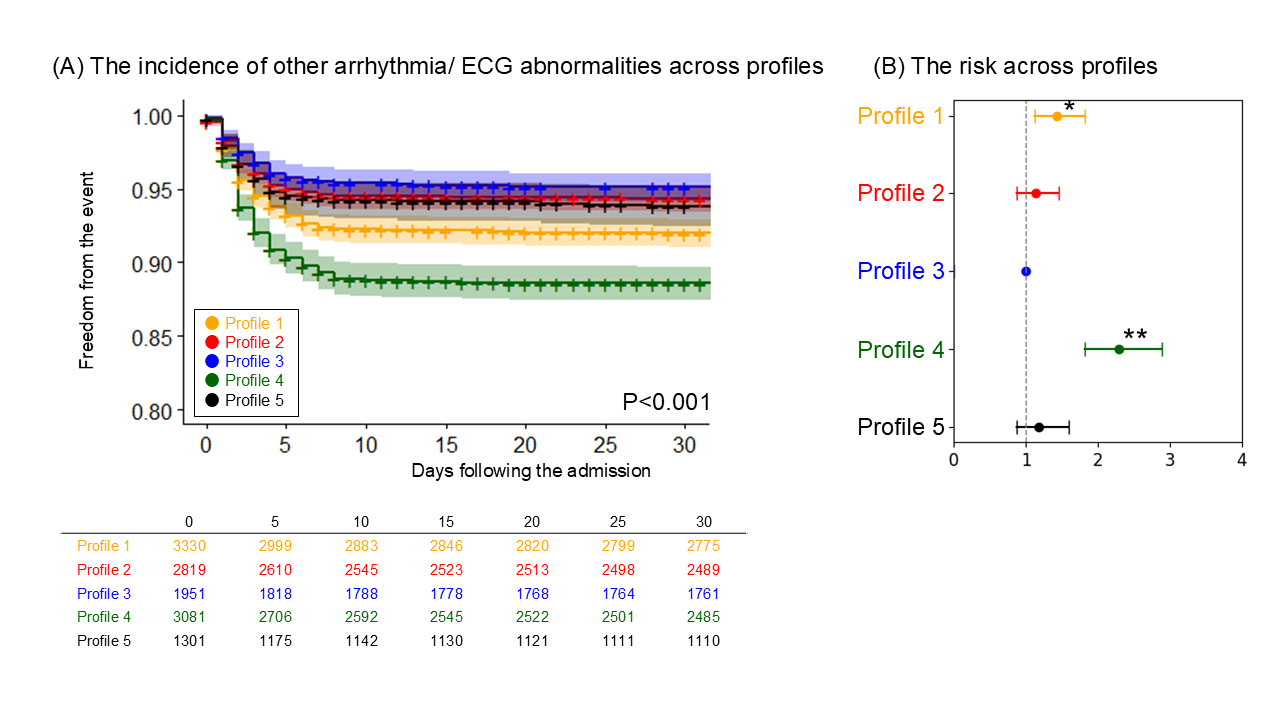


(A) The Kaplan-Meier curve. (B) Plot of hazard ratios adjusted for age and sex, with 95% confidence intervals.

ECG, electrocardiogram. Asterisk indicates statistical significance (*P<0.05, **P<0.001).

**Supplementary Table 1. The Comparison of Arrhythmia Subcategories across Profiles**

|  | Profile 1  (n=260) | Profile 2  (n=157) | Profile 3  (n=94) | Profile 4  (n=344) | Profile 5  (n=78) | P-value |
| --- | --- | --- | --- | --- | --- | --- |
| Bradyarrhythmias, n (%) | 96 (37) | 67 (43) | 36 (38) | 126 (37) | 25 (32) | 0.574 |
| Supraventricular arrhythmias other than AF/ AFL, n (%) | 35 (13) | 9 (6) | 7 (7) | 29 (8) | 13 (17) | 0.024 |
| Ventricular arrhythmias, n (%) | 45 (17) | 28 (18) | 15 (16) | 65 (19) | 10 (13) | 0.760 |
| Other arrhythmias, n (%) | 71 (27) | 45 (29) | 31 (33) | 104 (30) | 26 (33) | 0.809 |
| ECG abnormalities, n (%) | 13 (5) | 8 (5) | 5 (5) | 20 (6) | 4 (5) | 0.994 |

AF, atrial fibrillation, AFL, atrial flutter; ECG, electrocardiogram

**Supplementary Table 2. Cumulative Incidence Freedom from the Each Clinical Event across Profiles**

|  | Cumulative incidence freedom from the event, % (95%CI) | | | | | |
| --- | --- | --- | --- | --- | --- | --- |
|  | **Profile 1** | **Profile 2** | **Profile 3** | **Profile 4** | **Profile 5** | **P-value** |
| SHS | 84.5 (83.3-85.8) | 88.5 (87.4-89.7) | 90.8 (89.5-92.1) | 80.1 (78.7-81.6) | 87.4 (85.6-89.3) | <0.001 |
| ACS/ myocardial injury | 98.8 (98.5-99.2) | 98.8 (98.4-99.2) | 99.2 (98.8-99.6) | 98.4 (98.0-98.9) | 99.0 (98.5-99.6) | 0.200 |
| HF/ LV dysfunction | 97.4 (96.9-98.0) | 97.8 (97.3-98.4) | 98.2 (97.6-98.8) | 96.3 (95.6-96.9) | 97.4 (96.6-98.3) | <0.001 |
| AF/ AFL | 94.2 (93.4-95.0) | 96.3 (95.6-97.0) | 97.2 (96.4-97.9) | 93.0 (92.1-93.9) | 95.9 (94.8-97.0) | <0.001 |
| Other arrhythmia/ ECG abnormalities | 92.0 (91.1-92.9) | 94.3 (93.5-95.2) | 95.1 (94.1-96.1) | 88.6 (87.4-89.7) | 93.8 (92.5-95.1) | <0.001 |
| Cardiorespiratory arrest | 99.4 (99.2-99.7) | 99.5 (99.3-99.8) | 99.6 (99.4-99.9) | 99.1 (98.8-99.5) | 98.7 (98.1-99.3) | 0.007 |
| 90-day mortality | 86.4 (85.2-87.5) | 90.7 (89.6-91.8) | 93.1 (92.0-94.3) | 88.1 (86.9-89.2) | 87.2 (85.4-89.1) | <0.001 |

ACS, acute coronary syndrome; AF, atrial fibrillation, AFL, atrial flutter; AIS, acute ischaemic stroke; CI, confidence interval; ECG, electrocardiogram; HF, heart failure; LV, left ventricular; SHS, stroke-heart syndrome

**Supplementary Table 3. Modified Rankin Scale at Ninety-Day across Profiles**

|  | Profile 1 | Profile 2 | Profile 3 | Profile 4 | Profile 5 | P-value |
| --- | --- | --- | --- | --- | --- | --- |
| Modified Rankin Scale (mean±SD)* | 3.17±1.88 | 2.67±1.86 | 2.49±1.76 | 2.89±1.93 | 3.06±1.89 | 0.002 |

SD, standard deviation. Asterisk indicates statistical significance (*P<0.05).
